# Biofilm-Forming Capacity and *fimH* Gene Prevalence Among Uropathogenic *Klebsiella* spp. in Gabon: Assessing the Link with Resistance to Third-Generation Cephalosporins and Aminoglycosides

**DOI:** 10.3390/antibiotics15080736

**Published:** 2026-07-30

**Authors:** Evrard Mayombo Ngoussou, Rolande Mabika Mabika, Raoul Ampa, Ornella Zong Minko, Léonce Fauster Ondjiangui, Franck Mounioko, Florian Mbadinga Mackanga, Fred Stecy Litchangou Bouka, Mundunge Mambu, Jean Fabrice Yala

**Affiliations:** 1 Laboratoire de Bactériologie, Unité de Recherche d’Analyses Médicales, Centre Interdisciplinaire de Recherches Médicales de Franceville, Franceville BP 769, Gabon; mayombo.n.evrard@gmail.com (E.M.N.);; 2 Laboratoire de Biologie Moléculaire et Cellulaire, Faculté de Sciences et Techniques, Université Marien Ngouabi, Brazzaville BP 69, Congo; 3 Laboratoire de Biologie Moléculaire et Cellulaire, Université de Sciences et Techniques Masuku, Franceville BP 067, Gabon; 4 Unité de Recherche d’Ecologie en Santé, Centre Interdisciplinaire de Recherches Médicales de Franceville, Franceville BP 769, Gabon; 5 Laboratoire d’Analyses Biomédicales, Hôpital d’Instructions des Armées Omar Bongo Ondimba, Libreville BP 20 404, Gabon; 6 Laboratoire National de Santé Publique, Libreville BP 10 736, Gabon

**Keywords:** *Klebsiella* spp., biofilm, *fimH* gene, antibiotic resistance, Gabon, urinary tract infections

## Abstract

Background/Objectives: Bacteria of the genus *Klebsiella* are opportunistic pathogens frequently responsible for severe urinary tract infections (UTIs). Biofilm formation is a critical virulence factor that facilitates bacterial persistence and promotes the dissemination of antimicrobial resistance. This study aimed to characterize the biofilm-forming capacity and the prevalence of the fimH adhesin gene among clinical *Klebsiella* isolates in Gabon, and to evaluate their association with resistance to third-generation cephalosporins and aminoglycosides. Methods: A total of 114 urinary isolates, including *Klebsiella pneumoniae* (*n* = 96), *Klebsiella oxytoca* (*n* = 7), and *Klebsiella aerogenes* (*n* = 11), were analyzed. Biofilm formation was assessed using the crystal violet staining method and quantified by spectrophotometry. The *fimH* gene was detected using conventional PCR. Susceptibility to cefotaxime, ceftazidime, gentamicin, and amikacin was determined using an automated system. Results: The study revealed that 81.58% of the clinical *Klebsiella* isolates were biofilm producers. Quantitative analysis showed a predominance of weak (43.86%) and moderate (26.32%) producers, while only 5.26% were categorized as strong producers. The *fimH* gene was detected in 95.61% of the isolates, reaching 100% prevalence in *Klebsiella pneumoniae*. Interestingly, non-biofilm-producing *Klebsiella* spp. isolates exhibited the highest resistance rates to 3GC (67.90% for both cefotaxime and ceftazidime), suggesting that resistance to this class may be independent of biofilm-forming capacity in this cohort. Conversely, the highest resistance rates to aminoglycosides were observed among strong biofilm producers, reaching 60% for gentamicin and 40% for amikacin. Conclusions: Uropathogenic *Klebsiella* spp. in Gabon exhibit a high capacity for biofilm formation, with resistance patterns appearing to be antibiotic-class dependent. These findings suggest that considering biofilm phenotypes could be relevant for microbiological surveillance to improve the understanding of UTI resistance dynamics.

## 1. Introduction

*Klebsiella* species represent major opportunistic Gram-negative pathogens that pose a significant clinical challenge, as they are responsible for a diverse range of infections such as pneumonia, bacteremia, and urinary tract infections in both hospital and community settings [1,2]. A global modeling study on the burden of bacterial infections and antimicrobial resistance (AMR) estimated that deaths associated with *K. pneumoniae* reached 790,000 worldwide in 2019. Furthermore, *K. pneumoniae* was identified as the leading cause of neonatal mortality, accounting for 124,000 deaths [3]. These pathogens are in perpetual evolution globally and across Sub-Saharan Africa, particularly in Central Africa. Sub-Saharan Africa reported the highest age-standardized mortality rate linked to these bacterial agents in 2019, with figures reaching 230 fatalities per 100,000 individuals [3].

*Klebsiella* spp. are consistently reported as the second most frequently isolated pathogens in UTIs worldwide [4,5,6]. Bacterial UTIs represent a major global public health challenge, affecting individuals of all ages and both sexes [7]. Historically, these infections were effectively managed with conventional antimicrobials, specifically third-generation cephalosporins (3GCs) and aminoglycosides, which are widely used in empirical therapy due to their bactericidal and broad spectrum activity [8,9]. However, the efficacy of these therapeutic options is currently compromised by the continuous rise and dissemination of AMR, significantly limiting clinical alternatives [10]. For instance, recent studies from diverse geographical regions have documented an alarming upward trend in resistance; resistance to 3GCs and aminoglycosides has increased sharply over the last decades, with rates reaching between 60% and nearly 90% in some populations [11,12].

Resistance to these critical antibiotics is mediated by various bacterial mechanisms, often determined by the acquisition of resistance genes, such as the modification of antibiotic targets, reduced membrane permeability, and the activation of efflux systems [13]. In addition to these mechanisms, the ability of bacteria to form biofilms, particularly those responsible for UTIs, promotes adherence to surfaces, bacterial persistence, and horizontal gene transfer (HGT) among bacterial cells, thereby facilitating the spread of AMR genes [14]. Biofilms represent organized microbial assemblies, which may include single or multiple species, encased in a self-synthesized matrix of extracellular polymeric substances primarily made of proteins, polysaccharides, and extracellular DNA [15]. Biofilm formation enhances bacterial resilience against antimicrobial agents and environmental stressors, playing a central role in pathogenicity [16]. Consequently, in *Klebsiella* spp., biofilm formation represents a major virulence determinant, conferring superior survival in adverse conditions and serving as reservoirs and niches for genetic exchange, which contributes to the dissemination of AMR [17]. Biofilm formation is estimated to be involved in approximately 60% of hospital-acquired infections and as many as 80% of all microbial diseases, according to reports from the National Institutes of Health and the Centers for Disease Control and Prevention [18].

The ability of *Klebsiella* spp. to develop biofilms extends to both inanimate objects, including catheters and other medical equipment, and biological surfaces, specifically the mucosal layers of the respiratory, urinary, and digestive tracts [19]. The dense extracellular matrix acts as a physical barrier that restricts the diffusion of antimicrobial agents, allowing embedded bacteria to survive concentrations up to 1000 times higher than those that will kill planktonic cells [20]. Furthermore, numerous studies highlight the link between biofilm-forming capacity and increased antibiotic resistance in *Klebsiella* spp. isolates. For example, research conducted in Iran demonstrated a significant association between multidrug resistance (MDR) and biofilm formation, with the prevalence of biofilm-producing isolates being markedly higher among MDR strains (51%) compared to non-resistant isolates (24%) [21]. In agreement with these observations, a study by Pramodhini et al. on 100 urinary strains showed that biofilm-positive isolates were more resistant to ampicillin (83.3%) and cefotaxime (73.3%) than biofilm-negative ones, which exhibited lower rates of 60% and 35% [22].

To better understand the mechanisms underlying biofilm formation and bacterial persistence, several studies have focused on the molecular determinants involved in *Klebsiella* spp. Several factors contribute to biofilm formation in *Klebsiella* spp., including the capsule, fimbriae and pili, iron metabolism, and interactions with other bacterial species [16]. Among these determinants, Type 1 fimbriae, generally expressed by *Klebsiella* spp. strains and mediated by the *fimH* gene, are pivotal for binding to host epithelial surfaces, specifically within the urinary bladder lining [23]. In uropathogenic *Enterobacterales*, these Type 1 pili are major virulence factors involved in initial adherence, colonization, and persistence within the urinary tract [24]. The fimH-mediated fimbrial structures interact specifically with mannosylated receptors on the surface of urothelial cells, such as uroplakins, allowing for stable, shear-force-dependent adherence. Positioned at the terminal end of the fimbrial structure, the FimH adhesin promotes host cell entry and the subsequent establishment of intracellular bacterial communities [23]. Moreover, the expression of the *fimH* gene, which encodes the FimH adhesin of Type 1 fimbriae, contributes significantly to the initiation, maturation, and stability of biofilms, strengthening bacterial persistence and tolerance to host defenses and antimicrobial treatments. High prevalence rates of the *fimH* gene have been reported globally. In Europe, *fimH* was found in 91.7% of *K. pneumoniae* isolates from hospitalized patients in Poland, highlighting that it is a highly prevalent adherence factor involved in virulence and biofilm formation [25]. In Asia, a multicenter retrospective study on carbapenemase-producing and colistin-resistant *K. pneumoniae* isolates identified *fimH* as the most common gene, present in 22 isolates (81.48%), emphasizing its major role in virulence and biofilm formation [26]. Finally, in Egypt, a study on *K. pneumoniae* isolates responsible for nosocomial bloodstream infections observed that 86.7% of *fimH*-positive strains produced biofilms, compared to only 41.7% of *fimH*-negative isolates. These results highlight a significant association between the presence of the *fimH* gene and biofilm-forming capacity in *K. pneumoniae* [27]. Thus, these findings illustrate the global importance of *fimH* as a key determinant of virulence and bacterial persistence through biofilm formation.

Given the interdependence between fimH-mediated biofilm formation and MDR status, continuous molecular and phenotypic surveillance is essential. However, despite recent advances, our understanding of the interactions between biofilm formation and AMR in UTI-causing bacteria remains limited on a global scale. Furthermore, the distribution of genes involved in biofilm formation among these pathogens remains insufficiently explored. While, the role of *fimH* has been extensively studied in uropathogenic *Escherichia coli*, its prevalence and link to the biofilm phenotype in *Klebsiella* spp. remain poorly documented, particularly in Central African countries where genomic and phenotypic data are still scarce. This gap motivated the present study, which aimed to characterize the biofilm-forming capacity and evaluate the prevalence of the *fimH* adhesin gene among clinical urinary *Klebsiella* spp. isolates in Gabon, with a specific focus on their susceptibility to third-generation cephalosporins and aminoglycosides.

## 2. Results

### 2.1. Phenotypic Biofilm-Forming Capacity of Klebsiella spp. Isolates

The qualitative assessment of biofilm formation among the 114 *Klebsiella* spp. isolates was performed using the crystal violet staining method in 96-well microplates (Table 1).

The results, summarized in Table 1, demonstrate a high prevalence of biofilm-forming isolates. Overall, 81.58% (93/114) of the tested *Klebsiella* spp. isolates were biofilm producers, whereas 18.42% (21/114) were non-producers. Species-specific analysis revealed that *K. oxytoca* exhibited the highest proportion of biofilm producers (85.71%), followed by *K. pneumoniae* (82.29%) and *K. aerogenes* (72.73%). However, these differences were not statistically significant (Fisher’s exact test, *p* = 0.78).

Quantitative analysis through spectrophotometric measurements allowed for the categorization of isolates based on the intensity of biofilm production (Table 2).

The data indicate that biofilm-forming capacity varies significantly across *Klebsiella* species (*p* = 0.047). Globally, the majority of isolates were classified as weak producers (43.86%), followed by moderate producers (26.32%) and non-producers (24.56%). Only a small fraction (5.26%) was identified as strong biofilm producers. Within the most represented species, *K. pneumoniae*, the isolates were predominantly weak (42.7%) or moderate (31.3%) producers. In contrast, the majority of *K. aerogenes* (54.6%) and *K. oxytoca* (42.9%) isolates exhibited weak production, with a significant proportion of non-producers (36.4% and 57.1%, respectively).

**Figure 1:**
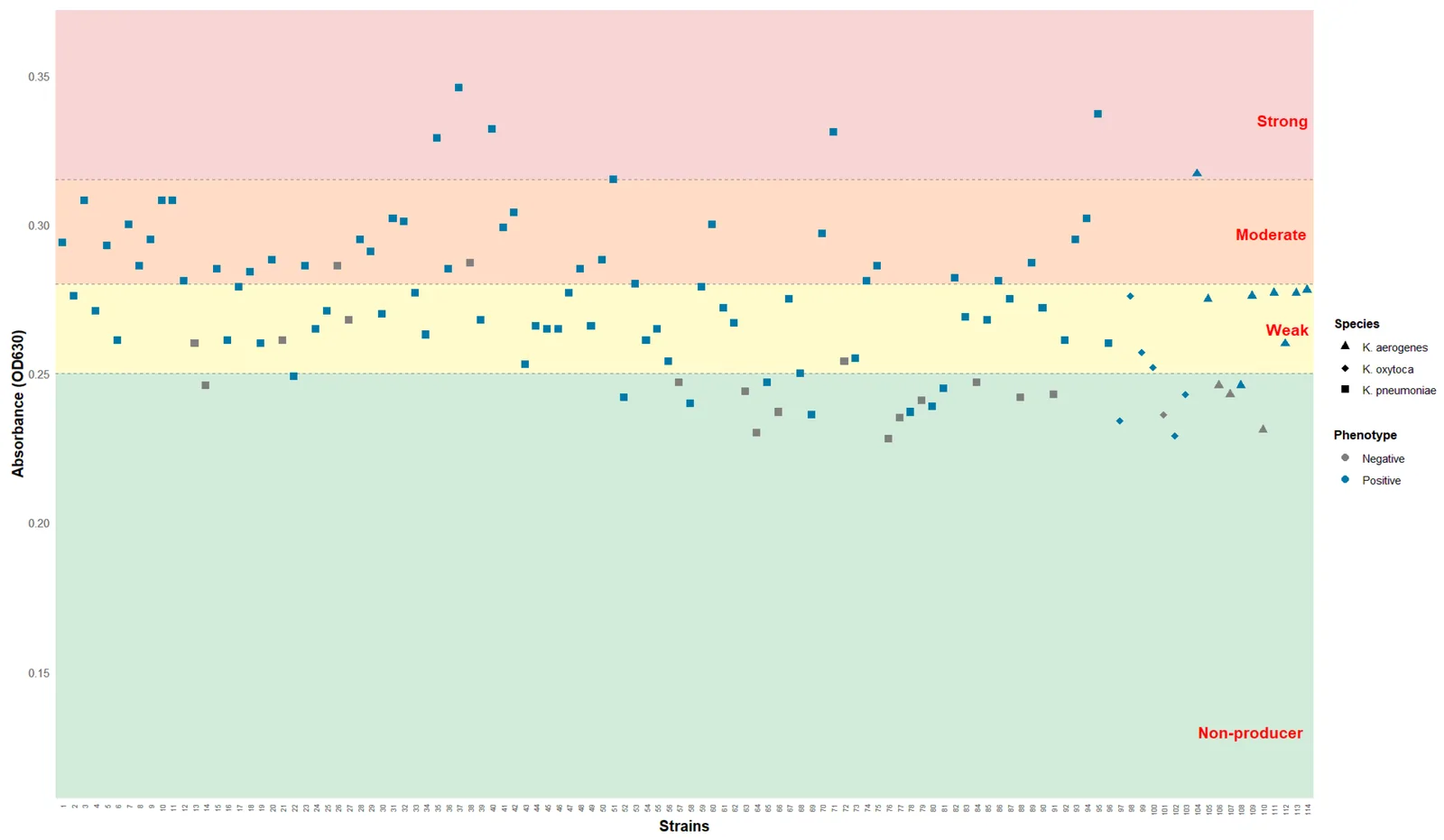
Distribution of *Klebsiella* spp. isolates based on qualitative assessment and biofilm formation categories.

The correlation between the two methodologies is detailed in Figure 1.

**Figure 2:**
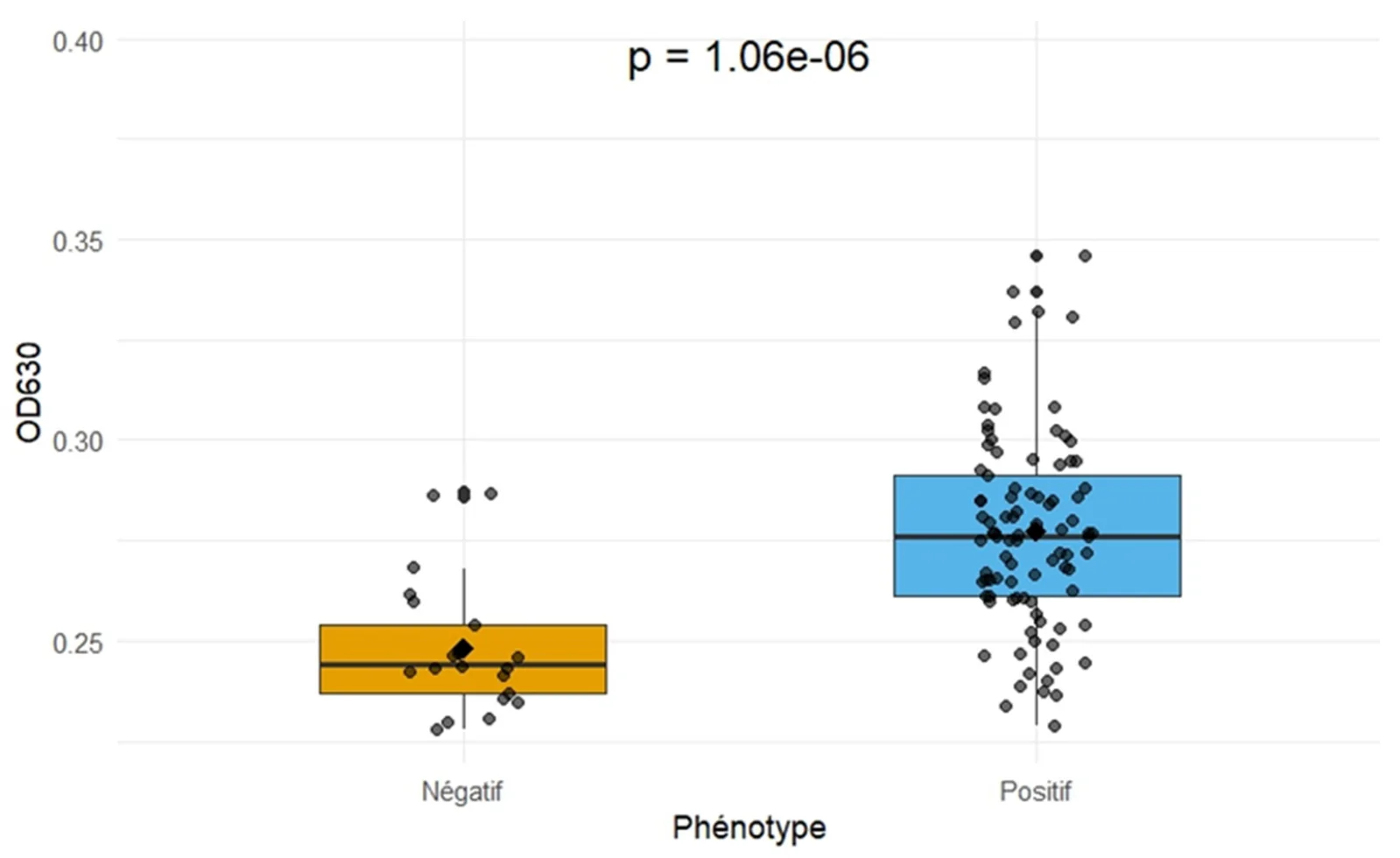
Box plot showing the correlation between qualitative and quantitative assessment methods.

Among the 21 isolates qualitatively identified as non-producers, 15 were quantitatively confirmed as non-producers, representing a 71.43% concordance. Among the 93 qualitatively positive isolates, 80 were quantitatively categorized as biofilm producers, reflecting an 86.02% concordance. This correlation was further validated by a Wilcoxon–Mann–Whitney test and visualized as a boxplot (Figure 2).

The boxplot suggests that qualitatively positive isolates reached a significantly higher optical density OD_630_ (median = 0.276) compared to negative isolates (median = 0.244). The variation was more pronounced in the positive group, as indicated by the box distribution. Statistical analysis confirmed that qualitatively positive isolates produced significantly higher biofilm densities than negative ones ($p=1.06\times 10^{-6}$). Although the absolute OD values remained within a narrow range between 0.240 and 0.350, this high statistical significance validates the distinction between the different biofilm-producing categories (Figure 2). This confirms that, under our experimental conditions, even subtle variations in absorbance reflect genuine differences in biofilm-forming capacity, justifying the use of the adapted thresholds for categorization.

### 2.2. Antimicrobial Resistance Mapping Relative to Biofilm Categories

**Figure 3:**
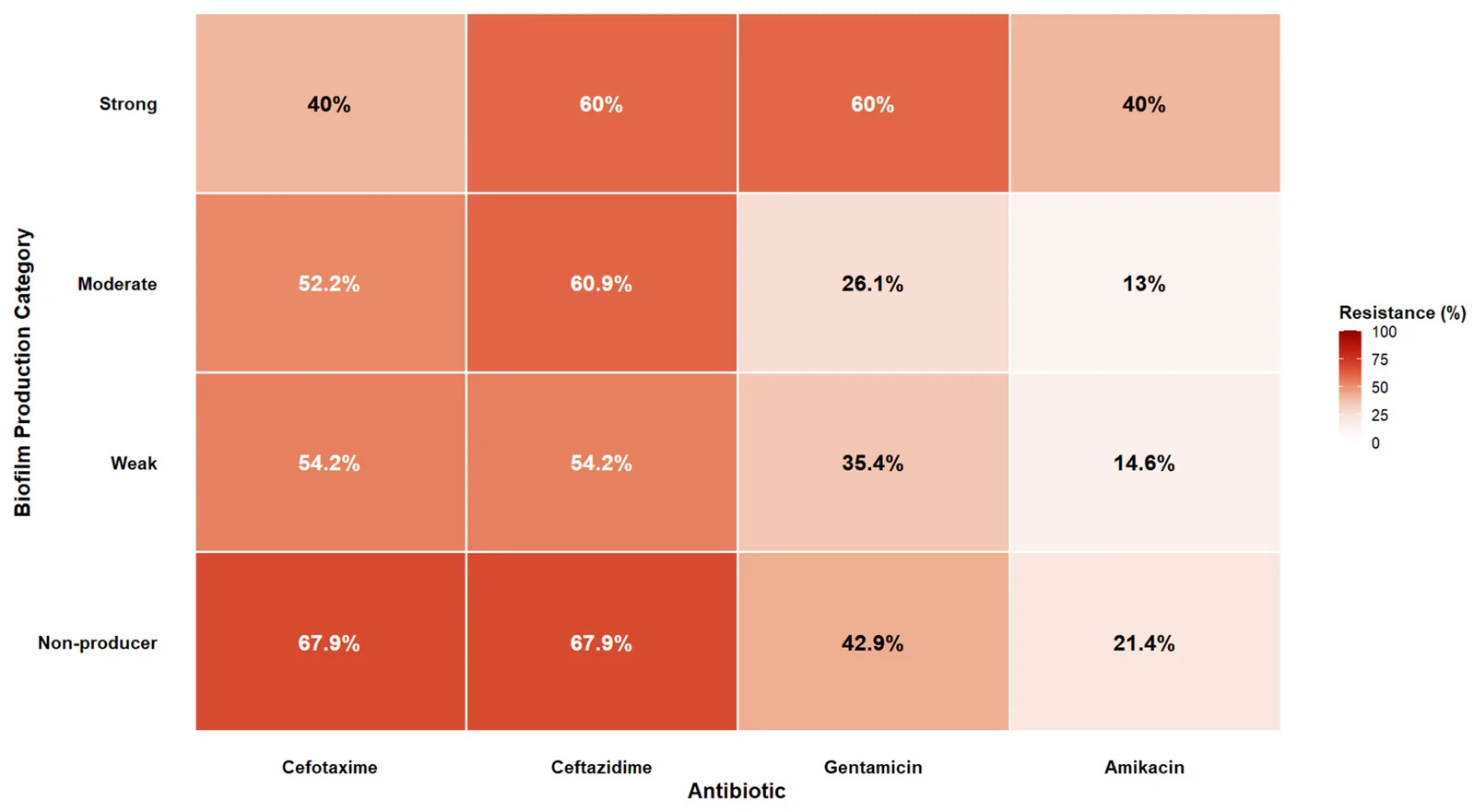
Antibiotic resistance profiles based on biofilm-forming categories.

Susceptibility profiles for third-generation cephalosporins (3GCs) and aminoglycosides were determined and correlated with biofilm categories (Figure 3).

Overall, *Klebsiella* spp. isolates exhibited high resistance rates to 3GCs (cefotaxime and ceftazidime) and lower resistance rates to aminoglycosides (gentamicin and amikacin). However, Fisher’s exact test showed that these differences were not statistically significant (all *p* > 0.05).

Interestingly, non-biofilm-producing isolates displayed the highest resistance rates to 3GCs (67.90% for both cefotaxime and ceftazidime; *p* = 0.526). Conversely, the highest resistance rates to aminoglycosides were recorded among strong biofilm producers, reaching 60% for gentamicin and 40% for amikacin (*p* = 0.451 and *p* = 0.395, respectively). Weak biofilm producers showed high resistance to 3GCs (54.20%) but relatively low resistance to aminoglycosides (35.40% for gentamicin and 14.60% for amikacin). Similarly, moderate producers exhibited high 3GC resistance (52.20% for cefotaxime and 60.90% for ceftazidime) but the lowest resistance rates to aminoglycosides (26.10% for gentamicin and 13.00% for amikacin). The specific biofilm-forming capacity (OD_630_) and the antibiotic resistance profiles for each *Klebsiella* isolate are detailed in Table S1 (Supplementary Materials).

### 2.3. Prevalence and Distribution of the fimH Gene Across Species and Biofilm Categories

**Figure 4:**
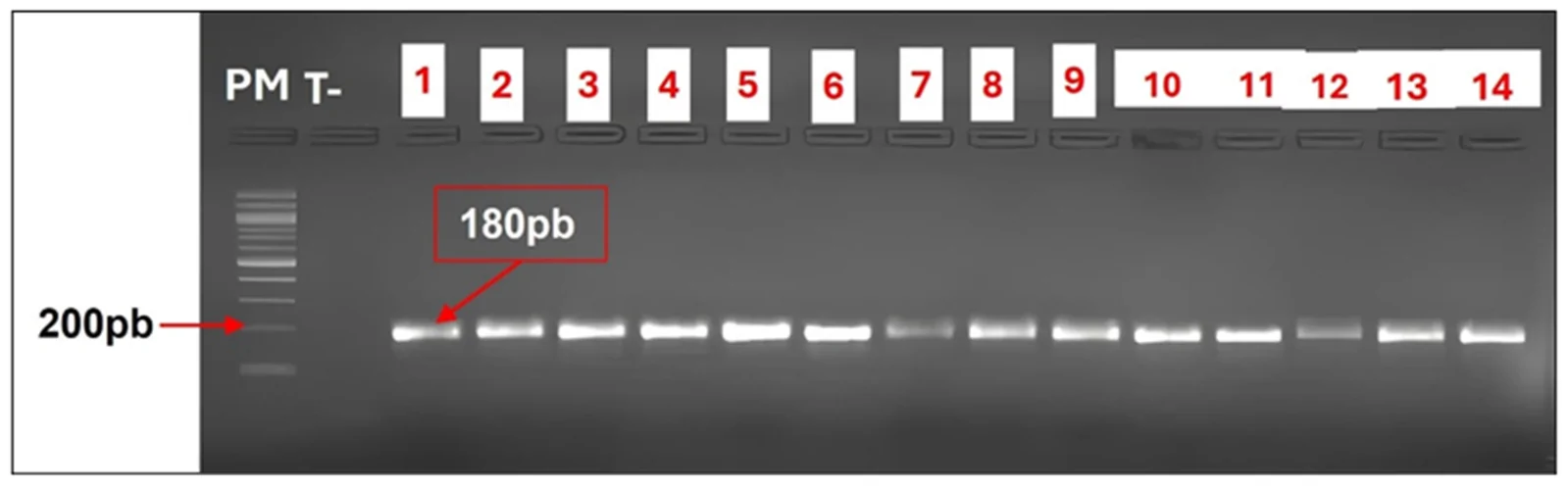
Agarose gel electrophoresis showing the detection of the *fimH* gene. Lane PM: DNA ladder; Lane T-: negative control; Lanes 1–14: *Klebsiella* spp. isolates.

The presence of the *fimH* gene was confirmed via agarose gel electrophoresis, identified by the characteristic 180 bp amplicons (Figure 4).

The molecular detection of *fimH* according to phenotypic biofilm production is presented in Table 3.

Overall, 95.61% (109/114) of the isolates were positive for *fimH*, while only 4.39% (5/114) were negative. The gene was detected in 95.70% of phenotypically positive biofilm producers and 95.24% of phenotypically negative isolates.

The distribution of the *fimH* gene among *Klebsiella* spp. isolates by species is presented in Table 4.

As shown in Table 4, the prevalence of the *fimH* gene varied across the isolates, being ubiquitous (100%) in *K. pneumoniae* and showing high frequencies in *K. oxytoca* and *K. aerogenes*.

The distribution of the *fimH* gene across the different biofilm production categories is presented in Table 5 below.

The prevalence of the *fimH* gene was high across all biofilm production categories, with no statistically significant association observed between the presence of the gene and the intensity of biofilm production (Fisher’s exact test, *p* = 0.56). Specifically, the results show that 100% (6/6) of the strong biofilm-producing isolates and 100% (30/30) of the moderate producers were positive for *fimH*. Furthermore, among the weak producers, 94% (47/50) carried the gene, while 6% (3/50) were negative. Similarly, within the non-biofilm-producing isolates, a high proportion was positive for the *fimH* gene at 92.86% (26/28).

## 3. Discussion

This study represents the first report from Gabon to evaluate the virulence of clinical *Klebsiella* spp. isolates through their biofilm-forming capacity (BFC). Our findings highlight a high prevalence of biofilm formation, with 81.58% of clinical isolates being qualitatively positive. This high frequency aligns with global trends, as *Klebsiella* species are well-recognized for their ability to colonize both abiotic and biotic surfaces, facilitating hospital persistence and the progression of chronic urinary tract and nosocomial infections [28,29]. The high proportion of biofilm-positive strains observed in this study confirms the central role of biofilms as a survival and resistance mechanism in this genus. Interestingly, *K. oxytoca* exhibited the highest biofilm-forming rate (85.71%), followed by *K. pneumoniae* (82.29%) and *K. aerogenes* (72.73%). While *K. pneumoniae* remains the most extensively studied species due to its frequent association with multidrug resistance (MDR) and hypervirulence, there is growing concern regarding *K. oxytoca* and *K. aerogenes*, which were formerly considered less virulent. Recent evidence suggests that these species possess biofilm-forming capabilities and virulence mechanisms comparable to *K. pneumoniae*, marking their emergence as significant clinical pathogens [30,31]. The inter-species variations observed in our study may stem from genetic diversity in adhesion systems, extracellular matrix regulation, or differential expression of structural genes involved in initial attachment [29]. However, these inter-species observations must be interpreted with caution. The significant discrepancy in the number of isolates, with *K. pneumoniae* (*n* = 96) being much more represented than *K. aerogenes* (*n* = 11) and *K. oxytoca* (*n* = 7), may limit the statistical power and the generalizability of these comparative findings.

Quantitative analysis revealed a heterogeneous distribution of biofilm production levels, dominated by weak to moderate producers. This profile is consistent with regional data; for instance, a study in Southwest Nigeria reported that 42.2% of *K. pneumoniae* isolates were moderate biofilm producers [32]. Similarly, research in Egypt found that 40.9% of isolates were moderate producers [33]. In contrast, only 5.26% of our isolates were categorized as strong producers, a result substantially lower than the 15–40% reported in the United States, Egypt, and Nigeria [32,33,34]. These discrepancies may be attributed to methodological and environmental factors. Protocol variations, including incubation conditions, culture media, and classification thresholds, significantly impact results [35,36]. Furthermore, our use of a 630 nm wavelength (outside the crystal violet peak absorbance of 570–590 nm) and 30% ethanol for solubilization, parameters that yield lower optical density ranges than standard protocols, likely explains the conservative proportion of ‘strong’ producers identified. Consequently, the categorization thresholds were adapted to these specific technical constraints to ensure an accurate phenotypic classification.

The correlation between qualitative and quantitative methods was high (86.02%), reinforcing the relevance of the crystal violet test as a reliable initial screening tool [37]. The Wilcoxon–Mann–Whitney test confirmed a significant difference in optical density between qualitatively positive and negative groups ($p=1.06\times 10^{-6}$), justifying the use of spectrophotometric quantification for more granular phenotypic analysis. Although the difference between the median OD of positive and negative isolates was numerically small, the high statistical significance (p<0.001) demonstrates that these values reflect distinct biological realities rather than experimental noise.

A critical finding of this study is the relationship between biofilm production and antibiotic resistance. Previous research has suggested varying correlations between these two phenotypes [22,38] The results of this study suggest that resistance to third-generation cephalosporins is particularly high among non-producers or weak producers, which contrasts with descriptions in previous studies. In their work, Pramodhini et al. reported that among 100 urinary isolates, resistance rates to ampicillin and cefotaxime were 83.3% and 73.3%, respectively, in biofilm-forming isolates, compared to only 60% and 35% in non-biofilm-producing isolates [22]. However, our findings suggest that the high resistance to 3GC observed among non-producers could be linked to the production of Extended-Spectrum Beta-Lactamases (ESBLs). This is consistent with the metabolic cost hypothesis: the high energy demand required for expressing complex resistance mechanisms (such as ESBL production) could potentially lead to a trade-off, reducing the resources available for biofilm matrix synthesis.

Conversely, we observed an apparent trend between the intensity of biofilm production and resistance to aminoglycosides. The highest aminoglycoside resistance rates were recorded among strong biofilm producers, reaching 60% for gentamicin and 40% for amikacin, compared to only 26% for gentamicin in moderate producers. These findings follow global trends. Indeed, several studies have demonstrated an association between biofilm-forming capacity and aminoglycoside resistance in *Klebsiella* spp. This phenomenon is exemplified by studies assessing the molecular characteristics of *K. pneumoniae* isolates, where a strong link was established between the development of biofilms and resistance to aminoglycosides. Specifically, amikacin resistance was found in 31.82% of biofilm-producing isolates, whereas no resistance was detected in non-producers. Similarly, gentamicin resistance reached 47.73% among biofilm producers, compared to 0% in non-producers [39]. This observed difference is consistent with the physical and chemical barrier effects exerted by the biofilm matrix. Aminoglycosides are polycationic molecules; as such, they are highly susceptible to electrostatic sequestration by the negatively charged components of the extracellular polymeric substance matrix, such as acidic exopolysaccharides and extracellular DNA [40]. Our data suggest that the increased density and complexity of the matrix in strong producers may contribute to trapping these antibiotics, limiting their diffusion and preventing them from reaching their intracellular targets. These observations support the view that biofilm-associated resistance is often an adaptive tolerance phenomenon driven by restricted penetration rather than a strictly genetic resistance. This might help explain why the impact of the biofilm is not uniform but antibiotic-class dependent, varying according to the specific mechanism of action and the molecular charge of the antimicrobial agent [15].

Molecular confirmation revealed a very high prevalence (95.6%) of the *fimH* gene among the isolates. This underscores the critical role of Type 1 fimbriae in uropathogenic *Klebsiella*. High *fimH* detection rates have been reported globally, including 87.50% in Iraq [41] and up to 100% in other clinical cohorts [42]. The near-ubiquity of the *fimH* gene in our isolates (96.51% of producers and 92.86% of non-producers) likely stems from their urinary origin. This gene, which is widely conserved across *Enterobacterales*, encodes Type 1 fimbriae adhesin, a crucial factor for attachment to urothelial cells [23,43]. However, its high prevalence among non-producers highlights that the presence of the genotype does not always translate into a biofilm-positive phenotype. This discrepancy may result from ‘phase variation’ or other transcriptional regulatory mechanisms where the gene remains silent. Consequently, while *fimH* is essential for initial adherence, the subsequent maturation of the biofilm and matrix production likely depend more on other determinants not explored here, such as *mrkA*, *pgaA*, or *wza* [41].

In conclusion, our results suggest that biofilm formation is not a universal predictor of antibiotic resistance in *Klebsiella* spp., but rather a specific modulator of resistance to certain classes, particularly aminoglycosides. This highlights the complex, multifactorial nature of antimicrobial resistance. These findings underscore the importance of integrating biofilm characterization into microbiological surveillance, especially for UTIs. However, this study has some limitations that should be noted. Our molecular characterization was focused solely on the *fimH* gene; while crucial for initial adherence, other key determinants such as the mrk operon (type 3 fimbriae), capsule-related genes (*wabG, wza*), or the pga operon also play significant roles in biofilm maturation and should be investigated in future studies. Furthermore, as an observational study, the associations reported here do not imply direct causation. Finally, another limitation concerns the methodology used for biofilm classification. Due to technical constraints, we employed a 630 nm wavelength and 30% ethanol for solubilization. This led to lower and more compressed absolute optical density (OD) values compared to the standard protocol (570 nm and 95% ethanol). While this internal classification is robust and consistent with our visual observations, the use of these modified cut-off values may limit direct quantitative comparisons with other studies strictly following the original Stepanovic criteria. Nevertheless, this work provides robust and essential data on the biofilm–resistance dynamics in an under-documented region and paves the way for targeted genomic investigations to improve the clinical management of *Klebsiella*-associated urinary tract infections in Central Africa.

## 4. Materials and Methods

### 4.1. Bacterial Strain Collection, Isolation, and Identification

A total of 114 clinical urinary isolates, comprising *K. pneumoniae* (*n* = 96), *K. oxytoca* (*n* = 7), and *K. aerogenes* (*n* = 11), were collected between April and December 2024 at the Omar Bongo Ondimba Military Teaching Hospital in Libreville, Gabon. These isolates were recovered from midstream urine samples submitted for cytobacteriological examination. A culture was considered positive when it showed monomicrobial or polymicrobial growth (limited to ≤ 2 organisms) with a bacterial load ≥10^5^ CFU/mL [44]. Isolates were preliminarily identified via conventional biochemical assays (API 20E; bioMérieux, Marcy-l’Étoile, France), whereas species confirmation and antimicrobial profiling were both carried out using the automated VITEK 2 Compact system (bioMérieux, Marcy-l’Étoile, France) using GN and AST-N441 cards, respectively. Although the system tested a broad panel of antibiotics, this study focused on third-generation cephalosporins (cefotaxime, ceftazidime) and aminoglycosides (gentamicin, amikacin), as these represent the primary therapeutic options for urinary tract infections in the Gabonese clinical setting.

### 4.2. Qualitative Assessment of Biofilm Formation (Crystal Violet Staining)

**Figure 5:**
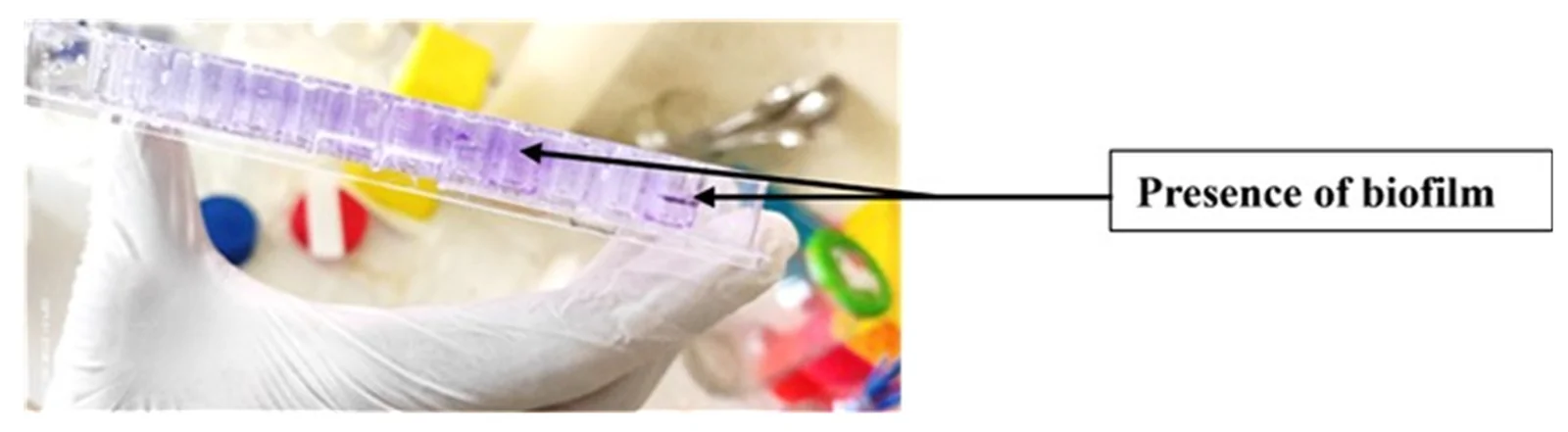
Visualization of biofilm formation on the microplate walls after crystal violet staining.

Biofilm formation was assessed using the standardized microtiter plate crystal violet staining method described by O’Toole [37], with minor modifications adapted to the study context. Briefly, 140 µL of Brain Heart Infusion broth (Scharlab S.L., Sentmenat, Spain) was dispensed into each well of a sterile 96-well microplate. Subsequently, 10 µL of a bacterial inoculum, pre-adjusted to a 0.5 McFarland turbidity level, was introduced into each well. Each strain was tested in duplicate. The microplates were incubated at 37 °C for 24 h. Following incubation, the wells were carefully decanted, rinsed with distilled water to remove planktonic cells, and air-dried at room temperature. The dried wells were then stained with 150 µL of 0.1% crystal violet (bioMérieux SA, Marcy-l’Etoile, France) for 10 min. After removing the excess dye and further drying, the presence of a visible purple film on the well walls was considered indicative of qualitative biofilm formation (Figure 5).

### 4.3. Quantitative Assessment of Biofilm Formation (D_630_ Measurement)

For the quantitative analysis, 150 µL of 30% ethanol was added to each pre-stained and dried well to solubilize the bound crystal violet. The microplates were incubated at room temperature for 10 min to ensure complete dissolution. Following the transfer of the resulting solution from each well into a fresh microtiter plate, absorbance was determined at 630 nm (OD_630_) using an Awareness Technology Inc. Stat Fax 3200 microplate reader (Awareness Technology Inc., Palm City, FL, USA).

### 4.4. Classification of Biofilm-Forming Capacity

Isolate classification based on biofilm production followed an adaptation of the scheme proposed by Stepanović et al. [45]. For this purpose, the threshold optical density (OD_c_) was established by adding three standard deviations to the average OD of the blank wells (uninoculated BHI broth). For this study, the $OD_{c}$ was established at 0.252. To account for the specific absorbance range obtained under our experimental conditions (30% ethanol and 630 nm reading), isolates were classified into four categories based on the following specific thresholds:

- Non-biofilm producers: OD≤0.252 ($OD\leq OD_{c}$);
- Weak producers: 0.253≤OD≤0.284 ($\approx 1.1\times OD_{c}$);
- Moderate producers: 0.285≤OD≤0.316 ($\approx 1.25\times OD_{c}$);
- Strong producers: OD>0.316 ($>1.25\times OD_{c}$).

### 4.5. Molecular Detection of the fimH Gene

Total genomic DNA was extracted from a bacterial suspension (prepared by resuspending colonies in 50 µL of sterile water) using the Quick-DNA Microprep Plus Kit (Zymo Research, Irvine, CA, USA) following the manufacturer’s instructions. The presence of the *fimH* gene, which encodes Type 1 fimbrial adhesin, was investigated using conventional PCR. The reaction was performed in a total volume of 20 µL, which contained 10 µL of AmpliTaq Gold^®^ 360 Master Mix 2X (Applied Biosystems, Thermo Fisher Scientific, Waltham, MA, USA) (containing hot-start Taq polymerase, $MgCl_{2}$, dNTPs, and optimized buffer), 1 µL of each primer (10 µM each), 2 µL of template DNA, and nuclease-free water. The primers used were: forward 5′-GCCAACGTCTACGTTAACCTG-3′ and reverse 5′-ATATTTCACGGTGCCTGAAAA-3′ [46]. Thermal amplification was performed using an initial 5-min denaturation step at 94 °C. This was followed by 35 cycles consisting of denaturation (94 °C for 30 s), primer annealing (43 °C for 30 s), and elongation (72 °C for 45 s), concluding with a final 5-min extension at 72 °C. The resulting amplicons were subsequently separated via 1.5% agarose gel electrophoresis and detected under UV illumination using a Quantum digital imaging platform. A band corresponding to the expected size of 180 bp was considered positive for the *fimH* gene.

### 4.6. Statistical Analysis

Statistical analyses were performed using R software (version 4.4.1) within the RStudio environment. Categorical variables were compared using Fisher’s exact test, which is appropriate for the small sample sizes in some subgroups. A *p*-value < 0.05 was considered statistically significant. Comparisons of biofilm-forming capacity between different groups were conducted using the Wilcoxon–Mann–Whitney test for non-normally distributed data.

## 5. Conclusions

This study provides the first comprehensive assessment of biofilm-mediated virulence among clinical *Klebsiella* spp. isolates in Gabon. Our findings reveal an alarming prevalence of biofilm-forming capacity, with *K. oxytoca* exhibiting the highest activity, followed by *K. pneumoniae* and *K. aerogenes*. At the molecular level, the nearly ubiquitous presence of the *fimH* gene reaching 100% prevalence in *K. pneumoniae*, suggests its potential importance in initial adherence and biofilm initiation.

The major contribution of this work is highlighting an apparent class-dependent association between biofilm formation and antimicrobial resistance. Based on our findings, biofilm production was not found to be a consistent predictor of resistance across all tested antimicrobials; instead, its impact appears to be class-dependent, which was observed for aminoglycosides but not for third-generation cephalosporins. While, resistance to aminoglycosides (gentamicin and amikacin) was observed to be higher, but not statistically significant, in strong biofilm producers, resistance to third-generation cephalosporins remained elevated regardless of biofilm-forming capacity, reflecting the coexistence of diverse genetic resistance mechanisms alongside phenotypic tolerance.

These observations suggest that biofilm formation may represent an adaptive mechanism associated with bacterial persistence and therapeutic failure within the Gabonese clinical context. Consequently, instead of routine clinical use, the targeted integration of phenotypic biofilm characterization into sentinel surveillance or specialized epidemiological studies should be considered. Such a research-oriented approach, complemented by molecular screening, could provide valuable insights that could help optimize therapeutic strategies for chronic or recurrent infections and improve the long-term management of *Klebsiella* infections in the region.

## Figures and Tables

**Table 1:** Qualitative assessment of biofilm production.

Biofilm Formation	*Klebsiella aerogenes*, *n* (%)	*Klebsiella oxytoca*, *n* (%)	*Klebsiella pneumoniae*, *n* (%)	Overall, *n* (%)	*p-*Value
Negative	3 (27.27)	1 (14.29)	17 (17.71)	21 (18.42)	0.78
Positive	8 (72.73)	6 (85.71)	79 (82.29)	93 (81.58)	

**Table 2:** Categorization of biofilm formation intensity of *Klebsiella* spp. isolates.

Biofilm Category	*Klebsiella aerogenes*, *n* (%)	*Klebsiella oxytoca*, *n* (%)	*Klebsiella pneumoniae*, *n* (%)	Overall *n* (%)	*p-*Value
Strong	1 (9.09)	0 (0.00)	5 (5.21)	6 (5.26)	
Moderate	0 (0.00)	0 (0.00)	30 (31.25)	30 (26.32)	0.047
Weak	6 (54.55)	3 (42.86)	41 (42.71)	50 (43.86)	
Non-producer	4 (36.36)	4 (57.14)	20 (20.83)	28 (24.56)	

**Table 3:** Distribution of the *fimH* gene according to biofilm formation status among the studied isolates.

Phenotypic Biofilm Detection	Total (*n*)	*fimH*-Positive, *n* (%)	*fimH*-Negative, *n* (%)
Negative	21	20 (95.24)	1 (4.76)
Positive	93	89 (95.70)	4 (4.30)
Total	114	109 (95.61)	5 (4.39)

**Table 4:** Distribution of the *fimH* gene among *Klebsiella* spp. isolates.

Species	Total (*n*)	*fimH*-Positive, *n* (%)	*fimH*-Negative, *n* (%)
*Klebsiella aerogenes*	11	7 (63.64)	4 (36.36)
*Klebsiella oxytoca*	7	6 (85.71)	1 (14.29)
*Klebsiella pneumoniae*	96	96 (100)	0 (0)

**Table 5:** Distribution of the *fimH* gene according to biofilm category.

Biofilm Category	Total (*n*)	*fimH*-Positive, *n* (%)	*fimH*-Negative, *n* (%)	*p-*Value
Strong	6	6 (100)	0 (0)	
Moderate	30	30 (100)	0 (0)	0.56
Weak	50	47 (94.00)	3 (6.00)	
Non-producer	28	26 (92.86)	2 (7.14)	

## Data Availability

The data presented in this study are available in the Supplementary Materials provided with the article.

## Supplementary Materials

The following supporting information can be downloaded at: https://www.mdpi.com/article/10.3390/antibiotics15080736/s1, Table S1: Phenotypic characteristics, biofilm formation (OD_630_), and antibiotic resistance profiles (Cefotaxime, Ceftazidime, Gentamicin, and Amikacin) of the tested *Klebsiella* spp. isolates.
