# Supplementary material for: Biofilm-Forming Capacity and fimH Gene Prevalence Among Uropathogenic Klebsiella spp. in Gabon: Assessing the Link with Resistance to Third-Generation Cephalosporins and Aminoglycosides
Source: Antibiotics (Basel). 2026 Jul 30;15(8):736. doi: 10.3390/antibiotics15080736 (PMC13510028; doi:10.3390/antibiotics15080736)
Supplement: Supplementary file 1 [file antibiotics-15-00736-s001.zip › antibiotics-4433834-supplementary.pdf]

| *Species* | Phenotype | OD630 | Catégorie | cefotaxime | ceftazidime | Gentamycine | Amikacine |
|---|---|---|---|---|---|---|---|
| *K. pneumoniae* | Positive | 0.294 | Moderate | R | R | S | S |
| *K. pneumoniae* | Positive | 0.276 | Weak | R | R | S | S |
| *K. pneumoniae* | Positive | 0.308 | Moderate | S | S | S | S |
| *K. pneumoniae* | Positive | 0.271 | Weak | S | S | S | S |
| *K. pneumoniae* | Positive | 0.293 | Moderate | R | R | S | S |
| *K. pneumoniae* | Positive | 0.261 | Weak | R | R | S | S |
| *K. pneumoniae* | Positive | 0.3 | Moderate | R | R | R | S |
| *K. pneumoniae* | Positive | 0.286 | Moderate | R | R | R | S |
| *K. pneumoniae* | Positive | 0.295 | Moderate | S | S | S | S |
| *K. pneumoniae* | Positive | 0.308 | Moderate | R | R | R | S |
| *K. pneumoniae* | Positive | 0.308 | Moderate | R | R | R | S |
| *K. pneumoniae* | Positive | 0.281 | Weak | R | R | R | S |
| *K. pneumoniae* | Negative | 0.26 | Weak | S | S | S | S |
| *K. pneumoniae* | Negative | 0.246 | Non producer | R | R | S | S |
| *K. pneumoniae* | Positive | 0.285 | Moderate | S | R | S | S |
| *K. pneumoniae* | Positive | 0.261 | Weak | S | R | S | S |
| *K. pneumoniae* | Positive | 0.279 | Weak | R | R | R | S |
| *K. pneumoniae* | Positive | 0.284 | Weak | R | R | R | S |
| *K. pneumoniae* | Positive | 0.26 | Weak | S | S | R | S |
| *K. pneumoniae* | Positive | 0.288 | Moderate | R | R | S | S |
| *K. pneumoniae* | Negative | 0.261 | Weak | R | R | S | S |
| *K. pneumoniae* | Positive | 0.249 | Non producer | R | R | R | S |
| *K. pneumoniae* | Positive | 0.286 | Moderate | R | R | R | R |
| *K. pneumoniae* | Positive | 0.265 | Weak | S | S | S | S |

| | | | | | | | |
|---|---|---|---|---|---|---|---|
| *K. pneumoniae* | Positive | 0.271 | Weak | R | R | R | S |
| *K. pneumoniae* | Negative | 0.286 | Moderate | | | | |
| *K. pneumoniae* | Negative | 0.268 | Weak | S | S | S | S |
| *K. pneumoniae* | Positive | 0.295 | Moderate | | | | |
| *K. pneumoniae* | Positive | 0.291 | Moderate | R | R | S | S |
| *K. pneumoniae* | Positive | 0.27 | Weak | R | R | R | S |
| *K. pneumoniae* | Positive | 0.302 | Moderate | R | R | S | R |
| *K. pneumoniae* | Positive | 0.301 | Moderate | R | R | S | R |
| *K. pneumoniae* | Positive | 0.277 | Weak | R | R | R | R |
| *K. pneumoniae* | Positive | 0.263 | Weak | R | R | S | S |
| *K. pneumoniae* | Positive | 0.329 | Strong | R | R | R | R |
| *K. pneumoniae* | Positive | 0.285 | Moderate | S | S | S | S |
| *K. pneumoniae* | Positive | 0.346 | Strong | S | S | S | |
| *K. pneumoniae* | Negative | 0.287 | Moderate | S | S | S | S |
| *K. pneumoniae* | Positive | 0.268 | Weak | S | S | S | S |
| *K. pneumoniae* | Positive | 0.332 | Strong | S | S | S | S |
| *K. pneumoniae* | Positive | 0.299 | Moderate | S | S | S | S |
| *K. pneumoniae* | Positive | 0.304 | Moderate | S | S | S | S |
| *K. pneumoniae* | Positive | 0.253 | Weak | R | R | R | S |
| *K. pneumoniae* | Positive | 0.266 | Weak | S | S | S | S |
| *K. pneumoniae* | Positive | 0.265 | Weak | R | R | S | S |
| *K. pneumoniae* | Positive | 0.265 | Weak | S | S | S | S |
| *K. pneumoniae* | Positive | 0.277 | Weak | | | | |
| *K. pneumoniae* | Positive | 0.285 | Moderate | S | S | S | S |
| *K. pneumoniae* | Positive | 0.266 | Weak | R | R | R | S |

| | | | | | | | |
|---|---|---|---|---|---|---|---|
| *K. pneumoniae* | Positive | 0.288 | Moderate | | | | |
| *K. pneumoniae* | Positive | 0.315 | Moderate | S | S | S | S |
| *K. pneumoniae* | Positive | 0.242 | Non producer | S | S | S | S |
| *K. pneumoniae* | Positive | 0.28 | Weak | S | S | S | S |
| *K. pneumoniae* | Positive | 0.261 | Weak | R | R | R | S |
| *K. pneumoniae* | Positive | 0.265 | Weak | S | S | S | S |
| *K. pneumoniae* | Positive | 0.254 | Weak | R | R | R | S |
| *K. pneumoniae* | Negative | 0.247 | Non producer | S | S | S | S |
| *K. pneumoniae* | Positive | 0.24 | Non producer | S | R | R | S |
| *K. pneumoniae* | Positive | 0.279 | Weak | S | S | S | S |
| *K. pneumoniae* | Positive | 0.3 | Moderate | S | R | R | S |
| *K. pneumoniae* | Positive | 0.272 | Weak | R | R | S | S |
| *K. pneumoniae* | Positive | 0.267 | Weak | R | R | S | S |
| *K. pneumoniae* | Negative | 0.244 | Non producer | R | R | S | S |
| *K. pneumoniae* | Negative | 0.23 | Non producer | R | S | S | S |
| *K. pneumoniae* | Positive | 0.247 | Non producer | R | R | R | S |
| *K. pneumoniae* | Negative | 0.237 | Non producer | R | R | S | S |
| *K. pneumoniae* | Positive | 0.275 | Weak | R | S | S | S |
| *K. pneumoniae* | Positive | 0.25 | Non producer | | | | |
| *K. pneumoniae* | Positive | 0.236 | Non producer | S | S | S | S |
| *K. pneumoniae* | Positive | 0.297 | Moderate | R | R | S | S |
| *K. pneumoniae* | Positive | 0.331 | Strong | S | S | S | S |
| *K. pneumoniae* | Negative | 0.254 | Weak | S | S | S | S |
| *K. pneumoniae* | Positive | 0.255 | Weak | R | R | S | S |
| *K. pneumoniae* | Positive | 0.281 | Weak | S | S | R | R |

| | | | | | | | |
|---|---|---|---|---|---|---|---|
| *K. pneumoniae* | Positive | 0.286 | Moderate | S | S | S | S |
| *K. pneumoniae* | Negative | 0.228 | Non producer | R | R | S | S |
| *K. pneumoniae* | Negative | 0.235 | Non producer | R | R | R | S |
| *K. pneumoniae* | Positive | 0.237 | Non producer | R | R | R | S |
| *K. pneumoniae* | Negative | 0.241 | Non producer | R | R | R | S |
| *K. pneumoniae* | Positive | 0.239 | Non producer | R | R | R | R |
| *K. pneumoniae* | Positive | 0.245 | Non producer | R | R | R | S |
| *K. pneumoniae* | Positive | 0.282 | Weak | R | R | S | R |
| *K. pneumoniae* | Positive | 0.269 | Weak | R | R | R | R |
| *K. pneumoniae* | Negative | 0.247 | Non producer | R | R | S | R |
| *K. pneumoniae* | Positive | 0.268 | Weak | R | R | R | R |
| *K. pneumoniae* | Positive | 0.281 | Weak | S | S | S | S |
| *K. pneumoniae* | Positive | 0.275 | Weak | S | S | S | S |
| *K. pneumoniae* | Negative | 0.242 | Non producer | R | R | S | S |
| *K. pneumoniae* | Positive | 0.287 | Moderate | | | | |
| *K. pneumoniae* | Positive | 0.272 | Weak | S | S | S | S |
| *K. pneumoniae* | Negative | 0.243 | Non producer | R | R | R | R |
| *K. pneumoniae* | Positive | 0.261 | Weak | R | R | R | R |
| *K. pneumoniae* | Positive | 0.295 | Moderate | | | | |
| *K. pneumoniae* | Positive | 0.302 | Moderate | | | | |
| *K. pneumoniae* | Positive | 0.337 | Strong | | | | |
| *K. pneumoniae* | Positive | 0.26 | Weak | | | | |
| *K. oxytoca* | Positive | 0.234 | Non producer | S | S | S | S |
| *K. oxytoca* | Positive | 0.276 | Weak | R | R | S | S |
| *K. oxytoca* | Positive | 0.257 | Weak | S | S | S | S |
| *K. oxytoca* | Positive | 0.252 | Non producer | R | R | S | R |

| | | | | | | | |
|---|---|---|---|---|---|---|---|
| *K. oxytoca* | Negative | 0.236 | Non producer | R | R | R | R |
| *K. oxytoca* | Positive | 0.229 | Non producer | R | R | S | S |
| *K. oxytoca* | Positive | 0.243 | Non producer | S | S | R | R |
| *K. aerogenes* | Positive | 0.317 | Strong | S | R | R | S |
| *K. aerogenes* | Positive | 0.275 | Weak | S | S | S | S |
| *K. aerogenes* | Negative | 0.246 | Non producer | S | S | S | S |
| *K. aerogenes* | Negative | 0.243 | Non producer | S | S | S | S |
| *K. aerogenes* | Positive | 0.246 | Non producer | R | R | R | S |
| *K. aerogenes* | Positive | 0.276 | Weak | R | R | R | S |
| *K. aerogenes* | Negative | 0.231 | Non producer | S | S | S | S |
| *K. aerogenes* | Positive | 0.277 | Weak | R | R | R | R |
| *K. aerogenes* | Positive | 0.26 | Weak | S | S | S | S |
| *K. aerogenes* | Positive | 0.277 | Weak | S | S | S | S |
| *K. aerogenes* | Positive | 0.278 | Weak | S | S | S | S |

Table S1: Phenotypic characteristics, biofilm formation (*OD630*), and antibiotic resistance profiles (Cefotaxime, Ceftazidime, Gentamicin, and Amikacin) of the tested *Klebsiella* spp. isolates.
